# Biotin Auxotrophy and Biotin Enhanced Germ Tube Formation in *Candida albicans*

**DOI:** 10.3390/microorganisms4030037

**Published:** 2016-09-21

**Authors:** Nur Ahmad Hussin, Ruvini U. Pathirana, Sahar Hasim, Swetha Tati, Jessica A. Scheib-Owens, Kenneth W. Nickerson

**Affiliations:** 1 School of Biological Sciences, University of Nebraska, Lincoln, NE 68588, USA; nahmadhussin@huskers.unl.edu (N.A.H.); ruvini@huskers.unl.edu (R.U.P.); sahar.hasim@gmail.com (S.H.); swetha.tati@gmail.com (S.T.); Jess.a.owens@gmail.com (J.A.S.-O.); 2 Department of Microbiology, University of Tennessee, Knoxville, TN 37996, USA; 3 Forrobin Inc., Buffalo, NY 14214, USA

**Keywords:** germ-tube formations, desthiobiotin, Lee’s medium, 7-keto-8-aminopelargonic acid (KAPA), upregulation of *BIO2*, *BIO3*, *BIO4*, mitochondrial *BIO2*, biotin starvation

## Abstract

Due to the increased number of immunocompromised patients, infections with the pathogen *Candida albicans* have significantly increased in recent years. *C. albicans* transition from yeast to germ tubes is one of the essential factors for virulence. In this study we noted that Lee’s medium, commonly used to induce filamentation, contained 500-fold more biotin than needed for growth and 40-fold more biotin than is typically added to growth media. Thus, we investigated the effects of excess biotin on growth rate and filamentation by *C. albicans* in different media. At 37 °C, excess biotin (4 µM) enhanced germ tube formation (GTF) ca. 10-fold in both Lee’s medium and a defined glucose-proline medium, and ca. 4-fold in 1% serum. Two biotin precursors, desthiobiotin and 7-keto-8-aminopelargonic acid (KAPA), also stimulated GTF. During these studies we also noted an inverse correlation between the number of times the inoculum had been washed and the concentration of serum needed to stimulate GTF. *C. albicans* cells that had been washed eight times achieved 80% GTF with only 0.1% sheep serum. The mechanism by which 1–4 µM biotin enhances GTF is still unknown except to note that equivalent levels of biotin are needed to create an internal supply of stored biotin and biotinylated histones. Biotin did not restore filamentation for any of the four known filamentation defective mutants tested. *C. albicans* is auxotrophic for biotin and this biotin auxotrophy was fulfilled by biotin, desthiobiotin, or KAPA. However, biotin auxotrophy is not temperature dependent or influenced by the presence of 5% CO_2_. Biotin starvation upregulated the biotin biosynthetic genes *BIO2*, *BIO3*, and *BIO4* by 11-, 1500-, and 150-fold, respectively, and *BIO2*p is predicted to be mitochondrion-localized. Based on our findings, we suggest that biotin has two roles in the physiology of *C. albicans*, one as an enzymatic cofactor and another as a morphological regulator. Finally, we found no evidence supporting prior claims that *C. albicans* only forms hyphae at very low biotin (0.1 nM) growth conditions.

## 1. Introduction

*Candida albicans* (*C. albicans*) is an important fungal pathogen of humans. As such, there has been intense interest in the virulence factors which make *C. albicans* such a versatile pathogen, and of these virulence factors the one which has garnished the greatest attention is the ability to interconvert between the yeast and hyphal morphologies. In his monumental work on Candida and Candidiasis, Odds [1] listed 34 chemical and environmental factors which favored filamentous forms and 25 factors which favored yeast/blastospore forms or suppressed hypha formation.

Media commonly used to produce *C. albicans* hyphae in vitro include: (i) Lee’s medium which contains eight amino acids [2]; (ii) serum [1,2]; (iii) buffered *N*-acetyl-d-glucosamine [3,4]; (iv) l-proline [5]; and (v) glucose-phosphate-proline (GPP) which may also have 2.5 mM *N*-acetyl-d-glucosamine (mGPP) [6], all at ≥37 °C. In addition, anaerobic growth gave hyphae at all temperatures tested from 25 °C to 37 °C [7].

Current research has focused on the signal transduction mechanisms and transcription factors which connect these external triggers with the induction of hyphae-associated genes [8]. Sudbery [8] distinguished nine positive and negative environmental cues. Farnesol and bacterial signaling molecules in the 3-oxo-homoserine lactone family were negative cues while the seven positive cues included: (i) growth in an embedded matrix or microaerophilic environment; (ii) low nitrogen; (iii) serum; (iv) >5% CO_2_; (v) l-methionine; (vi) *N*-acetylglucosamine; and (vii) a neutral pH or the absence of an acidic pH.

However, there are still many unanswered questions regarding the identity of the hyphal triggers, like which component(s) of Lee’s medium and serum actually induce hypha formation. These questions persist despite evidence that for serum both glucose [9] and bacterial peptidoglycan-like components [10] are part of the answer. Thus, we believe that some important hyphal triggers are still unknown or at least under-appreciated. Throughout, we will distinguish between a hyphal trigger, which by itself can initiate hyphal development, and an enhancer, which improves the effectiveness of a hyphal trigger.

*C. albicans* is a biotin auxotroph [1] and our previous work on biotinylated histones in *C. albicans* [11] showed that the cell yields for *C. albicans* in a biotin-free glucose-phosphate-proline (GPP) medium through three growth cycles (ca. 20 buddings) were ca. 60%, 40%, and 20%, respectively, of those achieved in a biotin replete medium. The presence of an internal biotin reservoir, possibly biotinylated histones, was indicated by the same decreasing cell yields being observed with and without added neutravidin in the biotin-free medium. Neutravidin is a strong biotin-binding protein.

The present paper studies the role of added biotin in germ tube formation at 37 °C. The interactions of biotin with yeasts date back to the discovery of biotin by Kӧgl and Tӧnnis [12]. They identified biotin as a growth factor obtained from eggs and yeast which promoted the growth of all baker’s and distiller’s yeasts tested, as well as a large number of other yeasts. Subsequently it was shown that *S. cerevisiae* needs biotin or the biotin analogs d-desthiobiotin, biocytin, or biotin-d-sulphoxide [13].

**Figure 1:**
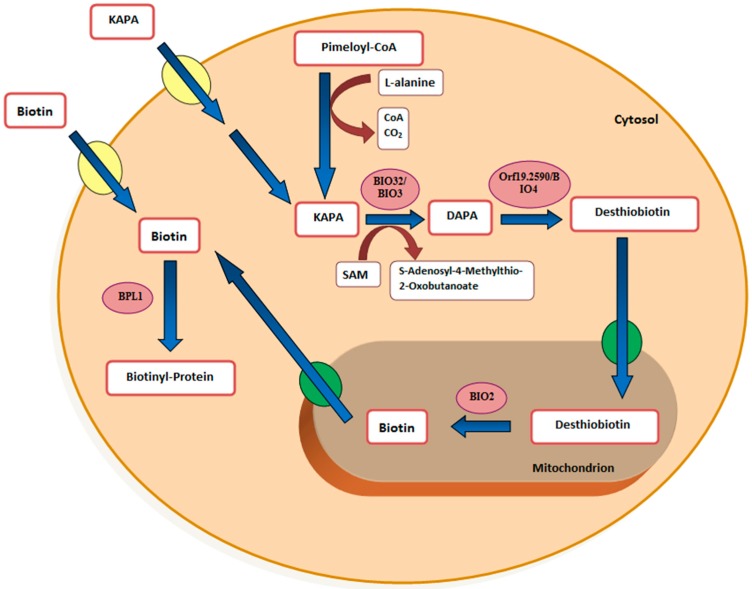
Diagram of proposed biotin biosynthesis pathway in *Candida albicans* based on a model available at the Candida Genome Database [18] and mitochondrial targeting predictions for *BIO32*, *BIO3*, *BIO4*, *BIO2*, and *BPL1*. This model assumes that biotin biosynthesis occurs in the cytoplasm except for the conversion of desthiobiotin to biotin by *BIO2*, which occurs in the mitochondrion. Biotin, desthiobiotin, and KAPA (8-amino-7-oxononanoate) could be taken up by two different transporters, which are *VHT1* and *HNM3/BIO5*, respectively. Note that the mitochondrial transporters for biotin uptake, desthiobiotin uptake, and biotin secretion may be different manifestations of the same transport system. This mitochondrion model is based on different roles of biotin and desthiobiotin in growth and filamentation.

Biotin biosynthesis is a variable trait in *S. cerevisiae* in that many strains only contain a partial biosynthetic pathway. Presumably the pathway was lost in a distant ancestor and then rebuilt by horizontal gene transfer, gene duplication, and neofunctionalization [14]. However, the last four steps are generally conserved: pimeloyl-CoA to 7-keto-8-aminopelargonic acid (KAPA) to 7,8-diaminopelargonic acid (DAPA) to desthiobiotin to biotin (Figure 1).

7-keto-8-amino-pelargonic acid and 7,8-diamino-pelargonic acid are also called 8-amino-7-oxononanoate and 7,8-diaminononanoate respectively. Thus, Phalip et al. [15] found that *S. cerevisiae* required an external supply of KAPA, DAPA, desthiobiotin, or biotin while two earlier precursors, pimelic acid and pimeloyl-CoA, would not suffice. *C. albicans* is also a biotin auxotroph [16] and Firestone and Koser [17] found that this biotin auxotrophy could be fulfilled by biotin, desthiobiotin, biocytin, or oxybiotin. Usually, 100 nM biotin is sufficient for optimal growth of *C. albicans* [1,6] and we observed equivalent growth rates and growth levels at 30 °C in GPP supplemented with 10 to 5000 nM biotin [11]. Significantly, *C. albicans* grown on excess (1–4 µM) biotin contained biotinylated histones even though cells grown on 100 nM biotin did not [11]. The present paper shows that 1–4 µM biotin also enhances germ tube formation in *C. albicans* and that the biotin present in both Lee’s Medium and serum is in part responsible for the ability of these media to achieve germ tube formation.

## 2. Materials and Methods

### 2.1. C. albicans Strains and Growth Conditions

*C. albicans* strain SC5314 was obtained from the American type culture collection, Rockville, MD and strain A72 was obtained from Prof. Patrick A. Sullivan, then at Otago Univ., Dunedin, New Zealand. The HLC52 (*efg1/efg1)*, JCK19 (*cph1/cph1*), and HLC54 (*efg1 cph1/ efg1 cph1*) mutants were from Prof. Gerald Fink and CR216 (*cdc35/cdc35*) was from Prof. Deborah Hogan (Table 1). Cells were grown overnight in 250 mL flasks with 50 mL of either Glucose-Salts-Biotin (GSB) or Yeast Extract-Peptone-Dextrose (YPD) media at 30 °C with aeration and were harvested by centrifugation at 5000 rpm for 5 min and washed three times with 50 mL of wash buffer (3.5 g KH_2_PO_4_, 4.1 g K_2_HPO_4_ in 1 L H_2_O, pH 6.5); samples were stored at 4 °C overnight for further use [6]. YPD consists of 10 g of yeast extract, 20 g of peptone, and 20 g of glucose per L. GSB is a minimal defined glucose-salts-biotin medium [6] and GPP is the defined glucose-phosphate-proline medium of Kulkarni and Nickerson [19]. We also used the defined glucose-ammonium-salts medium of Yamaguchi [20].

Cells were prepared for growth experiments by diluting the overnight culture in 50 mL of YPD, GPP, or GSB to an OD_600_ of 0.1 (1 × 10^6^ cells per mL). All OD_600_ values were recorded on a Spectra Max Plus microplate reader (Molecular Devices, Sunnyvale, CA, USA). Cells were tested in vitro with 2.5 mM *N*-acetylglucosamine at 37 °C [6] to be sure that their germ tube forming ability was close to 100%.

### 2.2. Analysis of Germ Tube Formation

*C. albicans* cells were inoculated at 1 × 10^7^ cells per ml (OD_600_ ~ 1.0) in prewarmed 25 mL flasks and supplemented with or without biotin (0–4 µM) or desthiobiotin (0–4 µM) and incubated with shaking (225 rpm) at 37 °C for 4 h. Every 30 min, a sample was analyzed microscopically. A total of 100–300 cells were counted in each experiment and the percentage of cells that had formed germ tubes was determined. Data from three independent experiments are presented. The effect of biotin in Lee’s medium was also observed in separate experiments conducted with and without l-proline [2]. Desthiobiotin was purchased from Santa Cruz Biotechnology, Santa Cruz, CA, USA while KAPA was from Cayman Chemical Co., Ann Arbor, MI, USA.

### 2.3. Analysis of Biotin Auxotrophy

*C. albicans* cells were grown at 1 × 10^6^ cells per ml in supplemented media (GPP, GSB) with or without biotin (0 or 100 nM) in 250 mL flasks. The cells for the first population were grown for 30 h at 30 °C or 37 °C (225 rpm). After 30 h, the cells were used as inoculum (1:100) and transferred to a fresh media supplemented with or without biotin (0 or 100 nM) and this population was counted as the second population. After 30 h growth, another 1:100 dilution gave the third population. For each population 5 mL samples were taken every 5 or 6 h, analyzed microscopically, and prepared for dry-weight measurement.

The dry-weight measurement was done using 5 mL samples that had been washed twice with distilled water (before drying). After measuring the initial weight of the weighing dish, the washed samples were dried in the drying oven for 6–8 h at 150 °C. Then, the samples were cooled in the desiccator. After constant measurement of the dry-weight of each sample, the data were recorded. Data obtained were from three independent experiments.

### 2.4. Effects of CO_2_ on Biotin Auxotrophy

Biotin deficient inocula were prepared by growing *C. albicans* SC5314 in 50 mL of biotin free glucose-salts medium pH (5.6) for 25 h at 30 °C. The cells were harvested, washed three times with KP buffer, and stored at 4 °C prior to use. The GS plates were prepared with Noble agar supplemented with 0, 5, 50, or 4000 nM biotin. Noble Agar was chosen to minimize the contaminating biotin provided by the agar. YPD agar plates were also used as controls. The inoculum was diluted to 6 × 10^2^ cells per ml so that a 100 µL cell suspension gave ca. 60 CFU/plate after they had been spread with 3–4 glass beads. Triplicate plates were incubated 4–5 days under four environmental conditions: (i) 30 °C; (ii) 37 °C; (iii) 30 °C with 5% CO_2_; and (iv) 37 °C with 5% CO_2_. Both 5% CO_2_ cultures were incubated in a dual chamber VWR 18401R incubator (VWR Scientific, Radnor, PA, USA). After 5 days all colony diameters were measured and averaged and then three colonies per plate were scraped off with a spatula and weighed.

### 2.5. Gene Expression Analysis

For gene expression analysis, a preculture of *C. albicans* grown in synthetic complete (SC) medium was reinoculated into SC medium with and without biotin until they reached mid log phase. The cells were harvested and total RNA were extracted using a phenol extraction method as described by Kohrer and Domdey [24] and then DNase treated with Turbo DNA-free kit (Invitrogen/Thermo Fischer Scientific) according to the manufacturer instructions. Complementary DNA was prepared using first strand cDNA using SuperScript^®^ III First-Strand Synthesis SuperMix for qRT-PCR (Invitrogen^™^) according to the manufacturer’s recommendation using oligo dT primers. Based on 1:1 ratio production of cDNA, a hundred ng of resulting sample was subjected to quantitative PCR using iQ^™^ SYBR^®^ Green Supermix in a Biorad iCycler iQ real time PCR detection system. Each cDNA preparation was normalized using *CaCDC36* as an internal control. The primers used for genes under study, *BPL1*, *BIO2*, *BIO3*, *BIO4*, *BIO32*, and *CDC36* in this study are listed in Table 2. Quantitative RT-PCR data were normalized in two steps as described previously [25] and analyzed using two-way ANOVA with the post Bonferroni comparison test using GraphPad Prism Version 5.02 (GraphPad Software, San Diego, CA, USA).

### 2.6. Mitochondrial Targeting Peptides (mTPs) Predictions

mTPs predictions on *BPL1*, *BIO2*, *BIO3*, *BIO4*, and *BIO32* were performed by using PSORT II [27] server, MitoFates [28] server, TPpred 2.0 [29] server and TargetP [30] server. Standard settings were used for all four predictors.

## 3. Results

### 3.1. Biotin Senhances Germ Tube Formation in both GPP and Lee’s Medium

**Figure 2:**
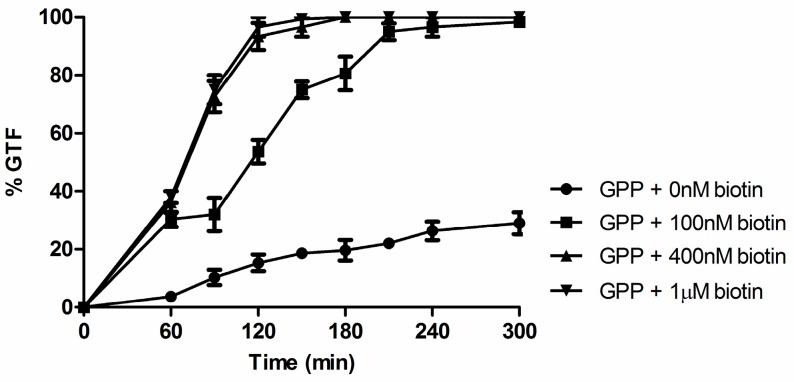
The germ tube formation (GTF) assays were conducted in glucose-phosphate-proline (GPP) (pH 6.5) containing variable levels of biotin at 37 °C with rotary agitation at 220 rpm. Cultures were observed for 5 h, and plotted as ●, no addition; ■, 100 nM biotin; ▲, 400 nM biotin; and ▼, 1 µM biotin. Values shown are the average of triplicate experiments ± SD.

**Figure 3:**
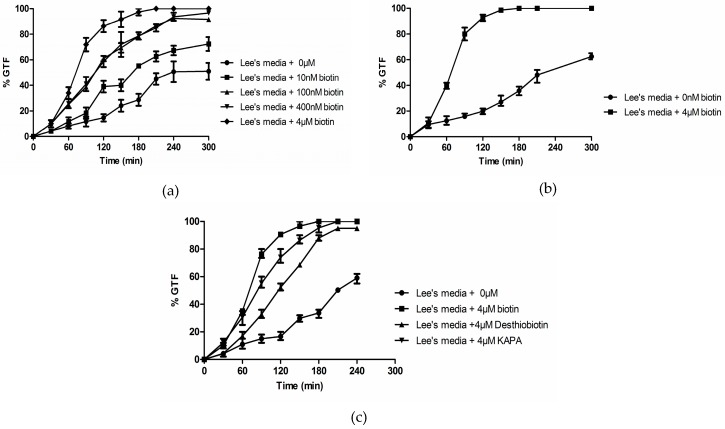
The germ tube formation (GTF) assays were conducted in Lee’s media containing variable levels of biotin at 37 °C with rotary agitation at 220 rpm. Cultures were observed for 4 to 5 h. (**a**) Dose-response for biotin. Symbols: ●, no addition; ■, 10 nM biotin; ▲, 100 nM biotin; ▼, 400 nM biotin; and ◆, 4 µM biotin. (**b**) GTF assays in Lee’s Medium prepared without l-proline. Symbols: ●, no addition; and ■, 4 µM biotin. (**c**) Desthiobiotin and KAPA also stimulate GTF. ●, no addition; ■, 4 µM biotin; ▲, 4 µM desthiobiotin; and ▼, 4 µM KAPA. Values shown are the average of triplicate experiments ± SD.

The chemically defined media commonly used to achieve germ tube formation (GTF) in *C. albicans* are remarkably diverse in their chemical composition. One feature which stands out is the concentration of biotin present, ranging from 20 µg/L in GPP up to 200 µg/L in RPMI 1640 and 1000 µg/L (4 µM) in Lee’s Medium [2], even though McVeigh and Bell [16] concluded that 1–5 µg/L biotin was sufficient for maximal growth. Significantly, both yeast nitrogen base and yeast carbon base contain only 2 µg/L biotin (Difco Manual) as does the glucose synthetic dextrose medium commonly used for *S. cerevisiae* [31]. Thus, the concentration of biotin in Lee’s medium is 500-fold higher than the 2 µg/L present in synthetic dextrose or yeast nitrogen base. Biotin (4 µM) stimulated GTF in both GPP (Figure 2) and Lee’s medium (Figure 3a).

In Lee’s medium we observed a dose-response relationship between % GTF and biotin (0 to 4 µM) such that the high biotin grown cells initiated GTF earlier and by 2 h had formed ca. 10-fold more germ tubes (Figure 3a). This relationship was also observed in a modified Lee’s medium from which l-pro, a known trigger for GTF, had been omitted (Figure 3b), and for Lee’s medium in which the biotin precursors desthiobiotin and KAPA replaced biotin (Figure 3c). These data show that the high concentration of biotin in Lee’s medium contributes to its ability to enhance GTF and that the presence of l-pro is not necessary for biotin to exert this activity.

### 3.2. Biotin Enhances Germ Tube Formation in Serum

**Figure 4:**
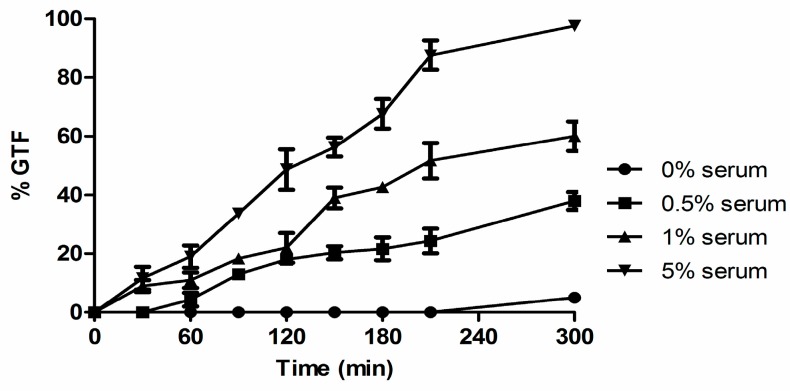
Effect of serum concentration on % GTF in *C. albicans*. Three times washed inocula were incubated at 37 °C with rotary agitation at 220 rpm. Cultures were observed for 5 h, and plotted as ●, no serum (water control); ■, 0.5% serum; ▲, 1% serum; and ▼, 5% serum. Values shown are the average of triplicate experiments ± SD.

Typically, concentrations of 10%–20% serum are used to induce GTF [1,32]. However, measuring the concentration of biotin in human serum is technically challenging [33]. It has been estimated to be ca. 150–700 ng/L [34] but RPMI 1640, a growth medium designed to replicate serum, contains 200 µg/L of biotin. To determine whether biotin contributes to the ability of serum to trigger GTF, we identified bovine serum concentrations that produced both high and low % GTF for 2 × 10^7^ cells at 37 °C after 5 h (Figure 4).

**Figure 5:**
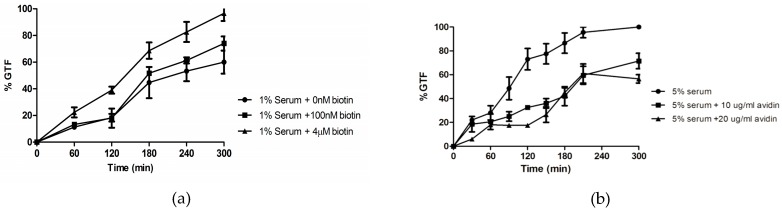
Effects of increased and decreased available biotin on GTF. Three times washed inocula were incubated at 37 °C with rotary agitation at 220 rpm. (**a**) Exogenous biotin increases serum induction of GTF. Cultures were observed for 5 h, and plotted as: ●, no additions; ■, 100 nM biotin; and ▲, 4 µM biotin. (**b**) Neutravidin reduces serum induction of GTF. Symbols: ●, no additions; ■, 10 µg/mL neutravidin; ▲, 20 µg/mL neutravidin. Values shown are the average of triplicate experiments ± SD.

Serum percentages as low as 0.5% achieved ca. 40% GTF and we chose 1% and 5% serum for low and high GTF, respectively (Figure 4). Significantly, the addition of 1–4 µM biotin doubled the % GTF in 1% serum (Figure 5a) while the addition of the biotin binding protein neutravidin (10–20 µg/mL) decreased the % GTF in 5% serum by 40% (Figure 5b).

These data support the idea that biotin is one of the factors in serum which enhances GTF. This emphasis on the importance of biotin does not, of course, mean that biotin is the sole trigger in serum. As a technical point, the cells used as inocula for these experiments on sera had been washed three times, as per our standard protocol. However, we also noted that the number of washes can be important. An experiment using sheep serum showed that the threshold for stimulating GTF was only 0.1% serum with the interesting feature that this low threshold could only be achieved with cells which had been washed six to eight times (Table 3). One possibility is that these extra washes act by removing cytoplasmic and membrane-bound farnesol which is still present after three or less washes [35].

### 3.3. Biotin Does Not Enhance Germ Tube Formation in N-Acetylglucosamine

**Figure 6:**
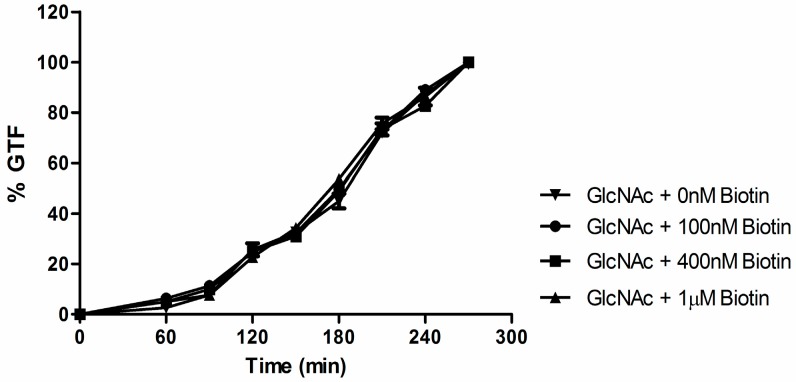
Germ tube formation at 2.5 mM *N*-acetylglucosamine (GlcNAc) is not affected by the addition of biotin. Three times washed inocula were incubated at 37 °C with rotary agitation at 220 rpm. Cultures were observed for 5 h, and plotted as: ▼, no addition; ●, 100 nM biotin; ■, 400 nM biotin; and ▲, 1 µM biotin. Values shown are the average of triplicate experiments ± SD. Virtually identical results were obtained using Yeast Extract-Peptone-Dextrose (YPD)-grown or defined Glucose-Salts-Biotin (GSB)-grown inoculum.

GTF can also be achieved without growth in a differentiation medium composed solely of *N*-acetyl-D glucosamine (GlcNAc), imidazole, and Mg/MnCl_2_ [4,6]. Biotin by itself could not trigger GTF in that it could not replace GlcNAc in the imidazole buffered differentiation medium and (up to 1 µM) it did not speed up the kinetics of GTF in the standard 2.5 mM GlcNAc differentiation medium (Figure 6).

### 3.4. Effects of Biotin on ∆efg1, ∆cph1, and ∆cdc35 Deletion Mutants

The previous series of experiments showed that biotin enhanced GTF in all growth media not already maximally stimulated by something else. However, as yet we do not know biotin’s mode of action. One approach to this question tests a series of mutants defective in hyphal formation to see if high biotin restored their hyphal forming ability. Accordingly, we examined the *∆efg1, ∆cph1*, and *∆cdc35* (adenylyl cyclase) deletion mutants of *C. albicans*. However, none of these mutants exhibited GTF in 2.5 mM GlcNAc, either with or without 4 µM biotin. These results are consistent with earlier reports that these mutants did not form germ tubes in Lee’s medium. Rocha et al. [23] showed that *C. albicans* mutants defective in adenylyl cyclase were unable to switch from yeasts to hyphae under all in vitro conditions analyzed, including Lee’s medium. Similarly, the hyphal defective mutants *cph1/cph1* [36] and *efg1/efg1* [22] were unable to form hyphae under all six of the hypha promoting conditions tested (not including Lee’s medium) but subsequently Doedt et al. [37] showed that the single mutants *efg1/efg1* and *cph1/cph1*, and the double mutant *efg1cphl/efg1cph1* did not form hyphae in Lee’s medium at 37 °C. These experiments collectively show that high biotin acts before adenylyl cyclase and the two transcription factors for hyphal associated genes, Efg1p and Cph1p.

### 3.5. Biotin Starvation Upregulates BIO2, BIO3, BIO4, and Biotin Protein Ligase BPL1

**Figure 7:**
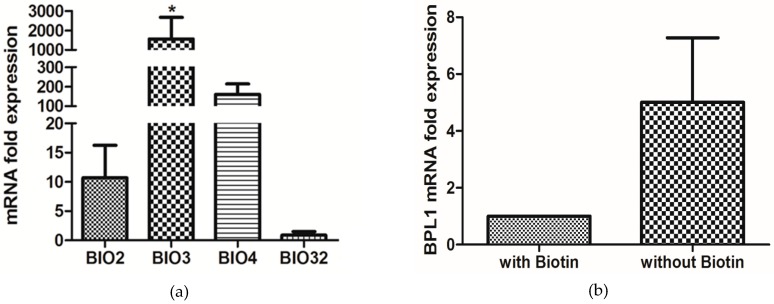
Relative expression of *C. albicans* predicted biosynthesis genes in synthetic complete (SC) medium under biotin starvation (no added biotin) relative to growth with 2 µg/L biotin (set as “1”) and normalized for the housekeeping gene *CDC36*. The experiment was done in three biological replicates and displayed as the mean ± SE. Significant upregulation of genes are marked by asterisk* (*p* < 0.05). (**a**) *BIO2*, *BIO3*, *BIO4*, and *BIO32*. (**b**) Biotin protein ligase, *BPL1*.

Regulation of the biotin biosynthetic genes in response to biotin starvation was explored in defined SC medium with and without 2 µg/L biotin (Figure 7). RT-PCR showed that the mRNAs for *BIO2, BIO3*, and *BIO4* increased 11-, 1500-, and 150-fold, respectively, in the biotin starved cells, while *BIO32* remained effectively unchanged (Figure 7a). The fact that *BIO32* remained unchanged is consistent with the suggestion by Fitzpatrick et al. [38] that *BIO32* is more likely to be involved in arginine or glutamate metabolism than in biotin synthesis. Interestingly, biotin protein ligase (*BPL1*) was also increased 5-fold in the biotin starved cells (Figure 7b).

### 3.6. Biotin Auxotrophy and Carbon Dioxide

**Figure 8:**
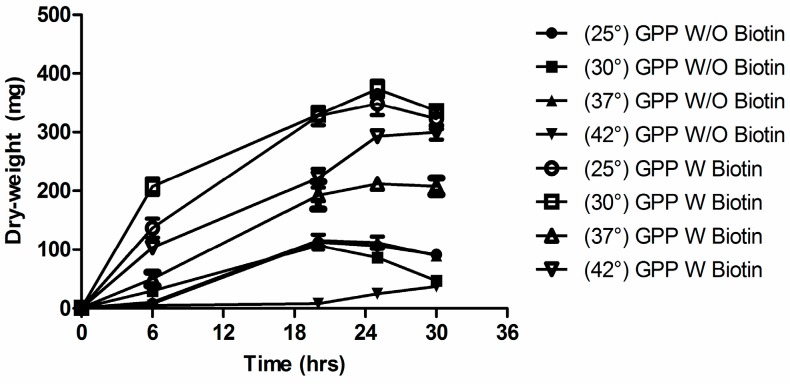
Lack of temperature dependence for the biotin auxotrophy. *C. albicans* SC5314 were grown for two cycles in GPP without biotin at 30 °C for 30 h and then washed three times. The graph shows cultures grown into GPP for another 30 h with or without the addition of biotin. Cultures were observed at different temperature, and plotted as (closed symbol—no addition of biotin): ●, 25 °C; ■, 30 °C; ▲, 37 °C; ▼, 42 °C; (open symbol—with 100 nM biotin): ○, 25 °C; □, 30 °C; △, 37 °C; ▽, 42 °C. After two washes, dry weights of the cells were determined. Values shown are the average of triplicate experiments ± SD.

*C. albicans* is a biotin auxotroph [16,17] and biotin is added routinely to all defined growth media for *C. albicans* [1]. We found that biotin, desthiobiotin, and KAPA were equivalent in fulfilling the biotin auxotrophy but pimelic acid was not (data not shown). Additionally, some vitamin requirements in bacteria and fungi are temperature dependent [39]. However, we found that the biotin auxotrophy in *C. albicans* is not temperature dependent; biotin was required for growth at all temperatures from 20 to 42 °C (Figure 8).

**Figure 9:**
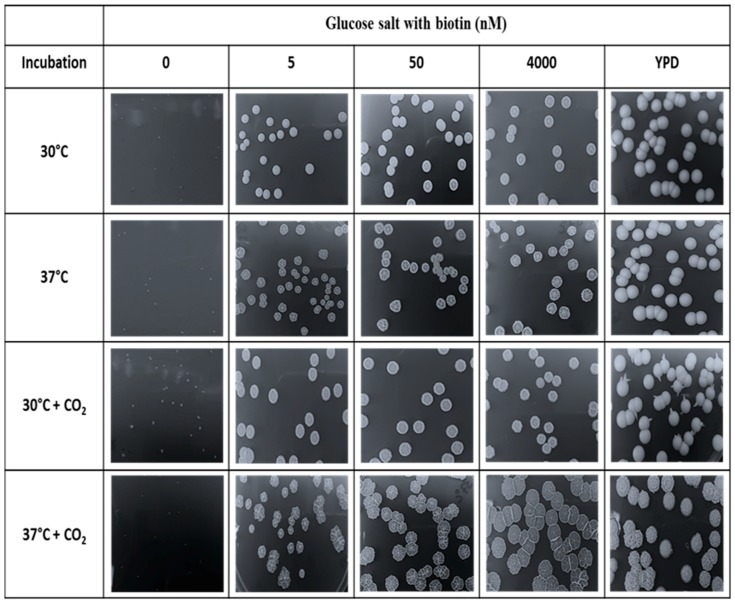
Colony morphology for *C. albicans* grown in YPD 2% agar or glucose-salts 2% Noble agar with 0, 5, 50, or 4000 nM biotin after 5 days incubation period at 30 °C or 37 °C with or without 5% CO_2_. Different magnifications were used to show colony morphologies. Noted that colony sizes were mentioned in Table 4.

We also examined the possible influence of 5% CO_2_ on biotin-dependent cell yields. This idea was tested because all biotin-containing enzymes have CO_2_ as either their substrate or product [40]. GS agar plates containing four levels of biotin (0–4 µM) along with YPD control plates were incubated under four conditions (30 °C and 37 °C both with and without 5% CO_2_) and colony diameter and dry weight were recorded after 5 days (Table 4). On these defined agar plates, the colonies were barely visible without the addition of biotin (Table 4 and Figure 9). However, with biotin the colony diameters and colony weights were only slightly smaller than those observed on the YPD plates (Table 4 and Figure 9).

Significantly, it did not matter whether the biotin supplemented plates contained 5, 50, or 4000 nM biotin; the colony diameters and mass were not statistically different (Figure 9). High biotin (4 µM) did not induce hyphal growth in these cases because the GS agar plates start at pH 5.6 and become more acidic as growth proceeds. Furthermore, 5% CO_2_ did not increase the colony diameter or mass except in one case (37 °C with 5% CO_2_) where the colony diameters with biotin (7 ± 0.3 mm) were twice as large (Table 4) because the colonies were predominantly filamentous and had spread. The filamentous nature of the colonies was determined by both microscopy and the wrinkled appearance of the colonies (Figure 9). Wrinkled colonies were observed for the 37 °C/CO_2_ grown cells regardless of whether the biotin supplemented plates contained 5, 50, or 4000 nM biotin (Figure 9) because 5%–10% CO_2_ also triggers GTF [41,42]. Finally, the short spikes seen emerging from the creamy, predominantly yeast colonies on YPD at 30 °C/CO_2_ (Figure 9) were also filamentous when observed by microscopy. Their presence likely results from the fact that the colony interior is anaerobic and anaerobically hyphal growth is expected at 25 °C and 30 °C as well as at 37 °C [7].

### 3.7. C. albicans 6713

Our data that high biotin stimulates GTF seems at variance with the work of Yamaguchi [20,43,44] who reported that GTF only occurred at low biotin (0.1–0.4 nM). That work assumes greater significance because it is cited as the scientific basis for the supposed health benefits of taking biotin pills to prevent/cure *C. albicans* infections. The growth conditions employed by Yamaguchi [20] were unusual in that the temperature shift was opposite to that usually employed to achieve GTF. Cells of *C. albicans* 6713 were scraped from Sabouraud 2% glucose agar at 37 °C and then inoculated into a glucose-ammonium salts medium (pH 5.5) at 29 °C [43] or 30 °C [20], even though it is now agreed [1,8] that an elevated temperature (37 °C) is required for all hypha-inducing conditions except growth in an embedded matrix.

We replicated the experimental conditions described by Yamaguchi [20] in all respects including the inoculum size. However, we were unable to demonstrate low biotin GTF with either *C. albicans* SC5314 or A72 at either 30 or 37 °C. *C. albicans* 6713 itself was no longer available. It is possible that high and low biotin GTF are separate and distinct phenomena because the levels of biotin employed are 10,000–fold different. It is also possible that low biotin GTF is characteristic of strain 6713 and not typical of other *C. albicans* strains. For instance, *C. albicans* 6713 could have had reduced levels of heat shock protein 90 (Hsp90) because strains engineered with reduced Hsp90 levels form hyphae in serum at 30 °C as well as 37 °C [45]. Finally, it is also possible that *C. albicans* 6713 would now be reclassified as *C. dubliniensis*, a closely related species known to produce fewer hyphae under all induction conditions tested [46]. These possibilities are not, of course, mutually exclusive.

## 4. Discussion

There are a great many chemical and physical signals for initiating germ tube formation and hyphal growth in *C. albicans*. These signals are important because yeast-mycelial dimorphism was the first virulence trait recognized in pathogenic fungi and as such it is somewhat perplexing that the actual germ tube triggers are still uncertain for Lee’s medium [2] and serum, two of the growth media most commonly used to get filamentous growth. This paper examined the physiological consequences of widely diverse levels of biotin (0–4 µM) on *C. albicans* with a view towards establishing high biotin as a factor that enhances germ tube formation (GTF). Biotin may eventually be shown to be a trigger for GTF, but at present, our data only prove that it is an enhancer of GTF. Three well studied conditions for GTF were shown to be biotin dependent: Lee’s medium, 1% serum, and GPP (Figure 2, Figure 3, Figure 4 and Figure 5). In response to high biotin (4 µM), *C. albicans* accumulates internal biotin reserves, forms biotinylated histones [11], and initiates germ tubes and hyphal growth. The hyphae produced during high biotin growth are physiologically normal in the sense that only 1–3 µM farnesol is needed to block GTF in Lee’s medium or RPMI 1640 for both *C. albicans* A72 and SC5314 [32].

*C. albicans*, like many yeasts, is a biotin auxotroph with an ability to accumulate internal, covalently attached biotin as a reserve for subsequent growth when biotin is limiting. Because of these internal reserves of biotin in *C. albicans*, claims for growth of *C. albicans* strain B 311-10 in minimal synthetic biotin-free medium [47] must be treated with caution because they used only a single passage of 30 h on the biotin-free medium. The rationale for *C. albicans* having an incomplete biosynthetic pathway for biotin (i.e., being able to utilize some biotin precursors as well biotin) is provided by the observation of Mock et al. [33] that biotin only accounts for half of the total avidin-binding substances present in human serum.

Despite being a biotin auxotroph, *C. albicans* SC5314 and WO-1 contain distinct biotin clusters including *BIO2*, *BIO3*, *BIO4*, and *BIO5* [38]. With regard to pathogenicity, the implication is that biotin or its precursors desthiobiotin and KAPA are available in the blood or another location where infection is occurring. Zakikhany et al. [48] catalogued the *C. albicans* genes which were significantly upregulated during infection; both *BIO2* and *BIO4* were upregulated ca. 2.5-fold. Similarly, *BIO2* was upregulated ca. 107-fold on shifting from low iron to high iron growth conditions [49]. Figure 1 illustrates biotin biosynthesis in *C. albicans*. For both yeasts, only *BIO2* is predicted to be mitochondrion-localized. Presumably *BIO2* has to remain mitochondrial because, as in *A. thaliana* [50], the biotin sulfur atom is obtained from an iron/sulfur cluster protein. *C. albicans* does not contain *BIO1* (pimeloyl-CoA synthase) or *BIO6*, the alternate method of synthesizing KAPA found in biotin prototrophic sake yeasts [51]. We anticipate that the isolation and characterization of mutants unable to respond to high biotin will elucidate biotin’s mode of action in stimulating GTF.

## 5. Conclusions

*C. albicans* is both a biotin auxotroph and a successful opportunistic pathogen. How is this possible? Part of the answer was shown previously [11] in that *C. albicans* contains an internal biotin reservoir, possibly in the form of biotinylated histones. We have now shown that high biotin (4 µM) is a previously unknown enhancer of germ tube formation (GTF) and its presence in both serum and Lee’s medium [2] is essential for hyphal formation. In particular, identifying biotin’s role in Lee’s medium solves a 40-year old puzzle regarding the actual trigger for GTF. The biotin precursors KAPA and desthiobiotin can replace biotin as both nutrient and as an enhancer of germination. This ability explains why *C. albicans* has a cluster of four genes (*BIO2–5*) encoding the latter half of the biotin synthetic pathway [38], implying that KAPA and/or desthiobiotin are present in blood or another location where infection occurs. The precise mechanism by which high biotin enhances GTF is as yet unknown, but in this regard, we are intrigued by the fact that the final step in biotin synthesis, *BIO2p* is localized to the mitochondria. Based on our findings, we suggest that biotin has two roles in the physiology of *C. albicans*, one being known as an enzymatic cofactor and another as a morphological regulator.

## Figures and Tables

**Table 1:** Lists of strains used.

*C. albicans* Strain	Genotype	Sources
SC5314	Wild-type	[21]
A72	Wild-type	ATCC * MYA-2430
HLC52	*ura3::*λ*imm434/ura3::*λ*imm434*	[22]
	*efg1::hisG/efg1::hisG-URA3-hisG*	
JCK19	*ura3::*λ*imm434/ura3::*λ*imm434*	[22]
	*cph1::hisG/cph1::hisG-URA3-hisG*	
HLC54	*ura3::imm434/ura3::imm434*	[22]
	*cph1::hisG/cph1::hisG efg1::hisG/efg1::hisG URA3::hisG*	
CR216	*ura3::*λ*imm434/ura3::*λ*imm434*	[23]
	*cdc35::hisG-URA3-hisG/cdc35::hisG*	

*: American Type Culture Collection.

**Table 2:** List of primers used for gene expression. Amplification was done using *CDC36* as the reference genes.

Gene Abbreviation	Primer Sequences (Forward -F and Reverse -R)	Source
*BIO2*	F: 5′-GCACCCAGAATCATTGCCAA-3′	This study
orf19.2593	R: 5′-ACTGCTCGTGTTCCTTCATG-3′	
*BIO4*	F: 5′-AGTAGCTCGGAGTGGATTGG-3′	This study
orf19.2590	R: 5′-TTAGAATGAGGGATGTTCGCA-3′	
*BIO32*	F: 5′-GTGGACGAGGATTATTTTGGGGAA-3′	This study
orf19.3567	R: 5′-TCCGTCTATTGTTCCCTTTCCA-3′	
*BIO3*	F: 5′-AAACTGGAGCCTGGGAAACT-3′	This study
orf19.2591	R: 5′-GGCGAACCCAAACACCTAAA-3′	
*BPL1*	F: 5′-GTTGAATGAGATCAGACGTGGA-3′	This study
orf19.7645	R: 5′-GCCATTGTCAACGTCCACTT-3′	
*CDC36*	F: 5′-GACCGTCCAGTATAAATCCACCAC-3′	Pendrak et al. [26]
	R: 5′-TCAAGACGGGCTCCACATTACTAT-3′	

**Table 3:** Effect of cell washings on serum-induced germ tube formation in *C. albicans.*

Percent Germ Tube Formation ^1^				
# Cell Washes	0.01	0.1	1.0	5.0% Serum
0	3	0	52	90
3	0	14	71	80
6	7	44	93	98
8	0	80	95	98

^1^ % GTF for *C. albicans* A72 after incubation for 4 h at 37 °C in the indicated % sheep serum. Serum diluent and cell washes used Kandiyohi distilled water [6]. Values indicated are the mean of two experiments which agreed within ± 10%. We previously showed [32] that pig, horse, sheep, and bovine sera gave virtually identical results.

**Table 4:** Influence of biotin and 5% CO_2_ on colony diameter ^1^ and colony weight after 5 days.

Glucose Salts with Biotin (nM)			
Incubation	0	5, 50, and 4000	YPD ^2^
30 °C	0.5 ^3^	4 ± 0.07 ^1^ (0.9)	6 ± 0.51 (1.4)
37 °C	0.5 ^3^	3.5 ± 0.15 (0.9)	6 ± 1.02 (1.4)
30 °C + CO_2_	1.0 ^4^	3.5 ± 0.05 (0.8)	5.5 ± 0.45 (1.4)
37 °C + CO_2_	0.5 ^3^	7 ± 1.55 (0.8)	6 ± 0.88 (1.4)

^1^ Colony diameter in mm (colony weight in mg). Values are the average of 7 replicates, using 3 biological replicates with 3, 2, and 2 technical replicates, respectively. Pictures of the colonies are shown in Figure 9. ^2^ Yeast Extract-Peptone-Dextrose (YPD) also serves as the 100% control for plating efficiency. All plates were spread with 100 µL of cells resulting in 60 ± 10 colonies per plate. ^3^ Colonies were too small to be scraped off and weighed. ^4^ Colonies could not be weighed because they had penetrated into the agar.
